# Utilization of immunotherapy as a neoadjuvant treatment in liver transplant recipients with hepatobiliary carcinoma

**DOI:** 10.3389/fonc.2026.1866870

**Published:** 2026-07-08

**Authors:** Maen Abdelrahim, Abdullah Esmail, Ashton A. Connor, Sudha Kodali, Ashish Saharia, David W. Victor, Caroline J. Simon, Yee Lee Cheah, Sunyoung S. Lee, Milind Javle, Ahmed O. Kaseb, Rafik Mark Ghobrial

**Affiliations:** 1 Section of Gastrointestinal Oncology, Houston Methodist Neal Cancer Center, Houston, TX, United States; 2 Department of Medicine, Weill Cornell Medical College, New York, NY, United States; 3 Department of Medicine, Faculty of Medicine, The University of Jordan, Amman, Jordan; 4 JC Walter Jr Center for Transplantation, Houston Methodist Hospital, Houston, TX, United States; 5 Department of Gastrointestinal Medical Oncology, University of Texas MD Anderson Cancer Center, Houston, TX, United States

**Keywords:** cholangiocarcinoma, hepatobiliary carcinoma, hepatocellular carcinoma, immunotherapy, liver transplant, Milan criteria, neoadjuvant

## Abstract

**Background:**

Hepatobiliary carcinoma (HBC) ranks as the sixth most prevalent cancer worldwide. Orthotopic liver transplantation (OLT) has emerged as a highly effective treatment option for early-stage HBC. Immunotherapies as neoadjuvant options are now being actively investigated in the transplant oncology era. This study reports our institutional experience in patients with HBC who had received immune checkpoint inhibitors (ICPI) prior to curative OLT.

**Methods:**

This retrospective cohort included 25 patients with HBC [17 with HCC and eight with cholangiocarcinoma (CCA)], who received ICPI prior to OLT at a single institution from January 2019 to December 2025. Graft rejection was assessed and reported along with the type of ICPI, tumor features, locoregional therapies (LRTs), and the timing of ICPI in association with OLT.

**Results:**

All 25 patients with HBC underwent OLT after neoadjuvant ICPI. A total of 19 patients were men, and six were women with a median age of 55 (interquartile range: 36–74) years at OLT. All patients received ICPI, including combinations of PD-1 inhibitors, PD-L1 inhibitors, and CTLA-4 inhibitors. Liver-directed/locoregional therapies were utilized in most cases, including transarterial chemoembolization (TACE), yttrium-90 (Y90), stereotactic body radiotherapy (SBRT), and radiofrequency ablation (RFA). The median washout period was 4.5 months. All patients responded to ICPI and achieved a safe and successful OLT. Most patients received tacrolimus plus mycophenolate as immunosuppressant (IS) therapy post-OLT, with 17 patients requiring additional IS, including prednisone or everolimus. One patient experienced immediate graft failure on the day of transplant, was made anhepatic, and was successfully retransplanted on the following day. Three patients experienced acute rejection during the first year post-transplant (two with liver rejection and one with kidney rejection following a deceased donor kidney transplant that was performed in conjunction with OLT).

**Conclusions:**

Our study highlights the potential of ICPI to achieve tumor downstaging prior to OLT in patients with HBC when given as a neoadjuvant therapy. In addition, this study illustrated the importance of timing for the administration of ICPI before OLT. Given the lack of conclusive evidence in this therapeutic area, these findings lay the groundwork for prospective trials to further examine the impact of ICPI in the pre-transplant setting.

## 1 Introduction

Hepatobiliary carcinoma (HBC) presents a significant global burden, with estimates from the year 2020 indicating that it ranks sixth in cancer incidence and third in cancer-related mortality (1). While some countries, primarily eastern Asia nations (China, Japan, and South Korea), have seen a decrease in liver cancer incidence, western countries, including the United States (US), have seen a stark rise in hepatobiliary cases (2). Liver cancer is now the second leading cause of premature cancer-related mortality (3). Hepatocellular carcinoma (HCC) accounts for over 80% of all diagnosed liver cancers, and despite the availability of therapeutic interventions, the 5-year survival ranges from 18%–43% (4, 5). The risk factors for primary liver cancers encompass a range of demographic indicators, including advanced age and sex, with men exhibiting a predominantly higher risk than women, alongside variations of risk associated with ethnicity. In multi-ethnic populations like the US, non-Hispanic White populations have a lower rate of incidence compared to Hispanic individuals, non-Hispanic Black individuals, and Pacific Island populations (6). In contributing to the development of hepatobiliary disease, some factors can be attributed to overall lifestyle, particularly nutritional habits. Preexisting clinical conditions including hepatitis B and C (HBV and HCV, respectively), alcohol use disorder, high-fat diets, biliary cirrhosis, exposure to food toxins such as aflatoxins, and inborn errors of metabolism such as alpha-1 antitrypsin deficiency, hemochromatosis, and Wilson’s disease also play an important role (7, 8). Exogenous risk factors are cigarette smoking, consumption of aflatoxin-contaminated food, and liver fluke infestation (8). Furthermore, while HCC associated with viral hepatitis has shown a decreased incidence, cases of HCC with hepatitis steatosis have been increasing in the US and represent a primary risk factor.

Orthotopic liver transplantation (OLT) has emerged as a highly effective treatment option for unresectable HCC in patients meeting the Milan criteria, which specifies a maximum tumor diameter of 5 cm for a single lesion or not more than three tumors, each less than or equal to 3 cm, without vascular invasion or extrahepatic metastases (9–13). However, in the US, the United Network for Organ Sharing (UNOS) exception points are available to patients within the Milan criteria or to those whose tumors have been successfully downstaged and are subsequently eligible for deceased donor allocation for liver transplantation. Though in cases of advanced-stage HCC where curative treatment options, including resection and liver transplantation, as well as locoregional therapies (LRTs) such as radiofrequency ablation (RFA) or microwave ablation and transarterial chemoembolization (TACE), are no longer viable, systemic treatment becomes necessary. Among the various therapies, neoadjuvant immune checkpoint inhibitors (ICPI) intervention has shown promising results in downstaging HBC and limiting tumor growth (14–17).

ICPI improves antitumor immunity by inhibiting the tumor-induced immune suppression of cytotoxic T-cells, leading to heightened immune activation. Recent studies indicate that immune-based therapies are effective in treating various solid tumors, demonstrating survival benefits and long-term disease control in conditions such as melanoma, lung cancer, and renal cell carcinoma (18).

ICPI represents the standard treatment for patients with unresectable or metastatic HCC, with atezolizumab–bevacizumab (Atezo/Bev) and durvalumab–tremelimumab (Durva/Treme) approved for first-line clinical use (19, 20). In patients with early-stage HCC, liver resection can lead to a cure; however, relapse rates may be as high as 70% within the initial 5 years following the procedure (21). It is essential to enhance the use of ICPI alongside curative treatments like resection or LRTs. Clinical studies demonstrate that PD-1 pathway inhibitors, with or without bevacizumab, improve relapse-free survival after liver resection compared to monitoring (22). The preoperative use of ICPI in patients with HCC has been explored in several early-phase studies assessing various treatment combinations, with an emphasis on safety and feasibility. Translational evidence indicates that neoadjuvant ICPI therapy may improve immune cell activation owing to the presence of the primary tumor *in situ*, leading to the expansion of T-cell clones and the reconstitution of exhausted tumor-specific lymphocytes (23).

In the clinical perspective, for over 10 years, sorafenib, a powerful oral multi-tyrosine kinase inhibitor (TKI) with antiproliferative and antiangiogenic properties, was the sole approved systemic first-line treatment for HCC. In the phase III SHARP trial (NCT00105443), sorafenib demonstrated a median overall survival (OS) of 10.7 months compared to 7.9 months in the best supportive care arm (24). In 2007, sorafenib received priority review from the US Food and Drug Administration (FDA) for the treatment of HCC due to the substantial survival benefit shown in the SHARP trial. At 10 years later, the phase III RESORCE (NCT01774344) trial evaluated the efficacy of the TKI regorafenib compared to placebo in HCC patients who had progressed on sorafenib treatment, demonstrating an improvement in overall survival of approximately 4 months relative to placebo (25). Regorafenib obtained FDA approval in April 2017, followed by the European Medicines Agency (EMA) approval later that year for second-line treatment of HCC (26).

The phase III CELESTIAL trial (NCT01908426) assessed cabozantinib versus placebo in patients with advanced HCC who had previously been treated with sorafenib (27, 28). In the second planned interim analysis published in July 2018, the median OS was reported as 10.2 months for cabozantinib and 8.0 months for placebo. Treatment with cabozantinib demonstrated a significantly longer OS compared to placebo (29). In May 2018, the FDA approved the application for cabozantinib for advanced HCC, followed by EMA approval for second-line HCC treatment in November 2018. Finally, the phase I/II CheckMate 040 trial (NCT01658878) and the phase II KEYNOTE-224 trial (NCT02702414), which evaluated nivolumab and pembrolizumab in patients with HCC previously treated with sorafenib, demonstrated similar overall response rates (ORR) of 18.2% for nivolumab according to the modified Response Evaluation Criteria in Solid Tumors (mRECIST) and 17% for pembrolizumab based on Response Evaluation Criteria in Solid Tumors (RECIST) 1.1 (30, 31).

This study reports our institutional experience and preliminary safety findings in patients with HBC (HCC and CCA) who received neoadjuvant ICPI-based therapy prior to curative OLT.

## 2 Methods

The data in this study were collected retrospectively from a carefully selected cohort, which included patients with primary liver cancer, HCC and CCA, who received ICPI prior to OLT. All surgeries took place at Houston Methodist Hospital from January 2019 to December 2025. The variety of ICPI and LRT that was applied to patients before transplantation and the post-transplant acute rejection, graft loss, HCC recurrence, and death were recorded.

OLT procedures were undertaken following clinical eligibility screening of patients with verified HCC and CCA diagnoses as confirmed by histopathological biopsy. For inclusion in this retrospective series, we identified patients who had achieved a minimum of 6 months of stable disease or tumor regression following neoadjuvant ICPI and whose tumor status had been maintained as unresectable, either due to tumor location or underlying liver disease, as documented by biopsy or cytology. Eligible patients had been reviewed by a multidisciplinary tumor board comprising gastrointestinal (GI), radiation and transplant oncologists, interventional radiologists (IR), hepatologists, and pathologists, with majority opinion guiding the assessment of tumor resection viability. Prior to transplant listing, patients had undergone full physical and psychological evaluations as part of the formal liver transplant evaluation process. This retrospective cohort was identified and reviewed through institutional records at Houston Methodist Hospital, and the study protocol was approved by the Houston Methodist Institutional Review Board (IRB ID: PRO00000587).

The participants in this series have continued regimented follow-up visits every 8 weeks to evaluate intervention response with imaging such as liver contrast enhancement in computed tomography (CT) or magnetic resonance imaging (MRI). Additional labs such as complete blood cell count, liver function tests [LFTs, aspartate aminotransferase (AST), alanine aminotransferase (ALT), and bilirubin], albumin test, and alpha-fetoprotein test (AFP) were scheduled and completed at these visits. The assessment of tumor response was made by utilizing the Modified Response Evaluation Criteria in Solid Tumors (mRECIST) (32).

From the statistical analysis standpoint, this is a descriptive study. All data were presented as median (IQR) for continuous measures and *n* (%) for categorical measures (**Table 1**). All analyses were performed using STATA version 17 (StataCorp., 2021, Stata Statistical Software: Release 17. College Station, TX: StataCorp LLC).

**Table 1:** Descriptive statistics: data are presented as median (interquartile range (IQR)) for continuous measures, and n (%) for categorical measures.

Total: *N* = 25	
Age: 55 (36.00–74.00)	
Sex	
Male	19 (76.00)
Female	6 (24.00)
Ethnicity	
Hispanic or Latino	6 (24.00)
Not Hispanic or Latino	19 (76.00)
Race	
Asian	5 (20.00)
Caucasian	18 (68.00)
Black	2 (08.00)
Stage of HBC at the Dx	
Stage I	5 (20.00)
Stage II	10 (40.00)
Stage IIIA	3 (12.00)
Stage IIIB	6 (24.00)
Stage IVA	0 (00.00)
N/A	1 (04.00)
Grade of HBC at the Dx	
Grade 1	5 (20.00)
Grade 2	9 (36.00)
Grade 3	7 (28.00)
Grade 4	0 (00.00)
N/A	4 (16.00)
Size of largest HBC lesion (cm) at the Dx	15 (0.3–15)
ECOG performance status	
0	12 (48.00)
1	12 (48.00)
2	1 (04.00)
3	0 (00.00)
Child–Pugh (CP) class	
A5	3 (12.00)
A6	3 (12.00)
B7	3 (12.00)
C10	0 (00.00)
C11	2 (08.00)
N/A	14 (56.00)
MELD from the Dx to OLT	
0–10	4 (16.00)
11–20	10 (40.00)
21–30	8 (32.00)
31–40	3 (12.00)
Comorbidities	
NASH and cirrhosis	6 (24.00)
ETOH and cirrhosis	1 (04.00)
HBV and cirrhosis	3 (12.00)
HCV and cirrhosis	5 (20.00)
HTN, BPH, and cirrhosis	1 (04.00)
HTN, DM, and cirrhosis	1 (04.00)
HTN and cirrhosis	1 (04.00)
Hemochromatosis and cirrhosis	1 (04.00)
Primary sclerosing cholangitis (PSC)	1 (04.00)
Secondary biliary cirrhosis	1 (04.00)
PRKACA gene rearrangement	1 (04.00)
Chronic cholecystitis	2 (08.00)
AFP (ng/mL)	04.00 (1.8–792.0)
Any treatment prior to ICPI	
Yes	7 (28.00)
No	18 (72.00)
Time for neoadjuvant (weeks): 52.00 (02.00–181.00)	

## 3 Results

In this retrospective study, 25 patients with HBC, including HCC and IHCCA/PCCA, underwent OLT following immunotherapy and locoregional treatments. The median age of the cohort was 55 years (range: 36–74), with a predominance of male patients (76%). Most patients identified as Caucasian (68%), followed by Asian (20%) and non-Hispanic Black (8%). Regarding ethnicity, 24% of patients reported as Hispanic or Latino.

At diagnosis, the HBC stage was primarily early to intermediate, with stage II and stage IIIA accounting for 40% and 12% of cases, respectively. The tumor grades varied, with grade 2 being the most prevalent (36%), and the largest lesion size ranged from 0.8 to 15 cm. The median model for end-stage liver disease (MELD) score before OLT was 21–30 in 32% of cases, reflecting advanced liver disease. Child–Pugh classifications indicated significant hepatic impairment, with 12% of patients in class B7 and 8% in classes C10/C11. Comorbid conditions were common and included non-alcoholic steatohepatitis (NASH, 24%), hepatitis C virus (HCV, 20%), and hepatitis B virus (HBV, 12%).

Immunotherapy regimens consisted primarily of PD-L1 inhibitors, with a minority of patients receiving PD-1, CTLA-4, or combination therapies. Locoregional treatments (LRT) included Y-90, TACE, SBRT, and RFA, administered either alone or in combination. The median waiting period from the initiation of neoadjuvant therapy to OLT was 52 weeks (range: 02.00–181.00). The pre-transplant alpha-fetoprotein (AFP) levels varied widely (median: 4.0 ng/mL; range: <1.8–792.0 ng/mL), reflecting tumor heterogeneity.

A pathological examination of the explanted specimens revealed multifocality in most cases (68%), with some presenting complete pathological response (ypT0). Tumor necrosis ranged from 10% to 100%, with extensive necrosis observed in a subset of cases. Lymphovascular and perineural invasion were noted in 36% and 16% of cases, respectively. Tumor grading in explanted specimens mirrored baseline findings, with moderate differentiation being the most common. Tumor staging post-OLT ranged from ypT0 to pT4, and fibrosis staging varied from bridging fibrosis to advanced cirrhosis.

Post-transplant immunosuppression regimens included combinations of tacrolimus with mycophenolate or everolimus and prednisone. Four participants (16%) had HCC recurrence during the first year post-transplant (diagnosed on days 41, 61, 221, and 361, respectively); no patient with CCA had a recurrence. One participant with CCA experienced immediate graft failure on day 0, was made anhepatic, and was successfully retransplanted on the following day. Three participants with HCC experienced acute rejection: one was a borderline rejection of the kidney on day 42 after liver and kidney transplant, one had a mild, focal T-cell-mediated acute rejection of the liver on day 162, and one had a moderate T-cell-mediated rejection of the liver on day 20 post-transplant. Thus far, four participants with HCC have died: three during the first year (one on day 61 due to disease recurrence and progression, one on day 66 due to multiorgan failure and septic shock, and one on day 342 due to a caval thrombus and metastatic HCC); one participant died on day 699 due to diffuse multifocal HCC recurrence with metastasis and opted for hospice care. None of the participants with CCA has died.

Patient 1 was a 66-year-old man with a history of hepatitis C and cirrhosis with ascites, jaundice, encephalopathy, Child–Pugh C, and a MELD-Na (Model for End-Stage Liver Disease—Sodium) score of 19. The patient was diagnosed with stage II, grade 3, poorly differentiated HCC. Within 4 months of diagnosis, the patient received a 4-month course of Atezo/Bev. The MRI result showed a significant response, with a decreased mass. At 1year after the initial diagnosis, the patient underwent an OLT. The patient received mycophenolate (500 mg) and tacrolimus (0.5 mg) twice daily (BID) for IS, with tacrolimus escalating from 2 to 4 mg and then decreasing back to 1 mg. The patient showed no clinical evidence of rejection.

Patient 2 was a 63-year-old man diagnosed with stage IA, grade 1, locally advanced HCC with LI-RADS 5 bilobar hepatic masses. During the following year, hepatic metastases, hepatic cirrhosis, fatty metamorphosis, and splenomegaly progressed. Therefore, the patient received three rounds of yttrium-90 (Y-90) therapy until lesion stabilization. The patient then underwent a partial hepatectomy. He received bridging therapy with nivolumab with approximately a 1-month washout period prior OLT. The patient also underwent TACE 2 weeks before transplantation. He underwent OLT 33 months after diagnosis. He received (200 mg) of mycophenolate and tacrolimus (0.5 mg) daily (escalating to 3 mg). The patient showed no clinical evidence of rejection.

Patient 3 was a 59-year-old man who presented with a history of chronic hepatitis B and elevated AFP levels. He was diagnosed with stage II, LI-RADS 4–5, multifocal HCC which was confirmed by wedge liver biopsy. Within a month of diagnosis, the patient started receiving lenvatinib and subsequently right-sided Y-90–2 months after diagnosis. He initially showed a significant response, but MRI after 10 months showed lesion progression. In response, the patient was switched to Atezo/Bev; at 3 months after Atezo/Bev administration, the patient underwent bilateral-sided TACE, with the left side showing a good response. Accordingly, a new regimen of cabozantinib was initiated 4 months prior to OLT. He underwent OLT 2.3 years after his initial diagnosis. The patient received (1,000 mg) of mycophenolate and (0.5 mg) of tacrolimus BID as IS. There was no clinical evidence of graft rejection.

Patient 4 was a 62-year-old man who presented to the hospital with abdominal pain and was found to have a 1.9 cm lesion compatible with HCC and a suspicious 1.4 cm new lesion. The patient had a medical history of hemochromatosis, cirrhosis, non-alcoholic steatohepatitis (NASH), and hemosiderosis. The patient had a 2.1-cm LI-RADS 5 lesion 2 months before starting immunotherapy. Lenvatinib was started 1 year and 7 months post-diagnosis but discontinued due to intolerance. Nivolumab began a month later and was shown to be well tolerated, but HCC progressed. He switched to Atezo/Bev for 2 months with a 2-month washout period prior to OLT, which he received 2.5 years after diagnosis. The patient received mycophenolate (500 mg) BID, prednisone (5 mg), and tacrolimus (3 mg) BID. At 1 year after OLT, no evidence of acute rejection or relapsed cancer was observed.

Patient 5 was a 68-year-old man who presented with stage IIIB HCC (cT4, cN0, and cM0) and had multiple bilobar lesions, including a 7.5-cm LI-RADS 5 lesion in segment 5/6. He started Atezo/Bev at 2 months post-diagnosis, which was discontinued after 15 months due to disease progression. The patient had undergone multiple Y-90 procedures. At 1 month after discontinuing Atezo/Bev, the patient started oral lenvatinib and had excellent tolerance. Imaging at 5 months pre-OLT showed residual disease in segment 4 and a stable LI-RADS 3 nodule in segment 8. The patient underwent OLT 2.5 years after the initial diagnosis. The patient received 500 mg BID of mycophenolate and 0.5 mg BID of tacrolimus, which was increased to 4 mg and then tapered to 0.5 mg. He developed chronic respiratory failure requiring tracheostomy, sustained bacteremia with septic shock, and had renal failure requiring dialysis. However, clinical graft rejection was not suspected or observed in our patient.

Patient 6 was a man aged 61 years who had previously been diagnosed with HCC related to hepatitis B and initially underwent resection, but recurrence required re-resection 3 years later. He received preoperative nivolumab and ipilimumab but experienced presumed ICPI-related hepatotoxicity (autoimmune hepatitis) that improved on prednisone. On day 361 after resection, imaging showed tumor recurrence with a new lesion, which was treated with RFA. At 3 months later, a 1-cm tumor near the previous recurrence was also treated with RFA. At 9 months after recurrence, a 2.4-cm tumor adjacent to the left portal vein was treated with TACE. Imaging later revealed a new 3.3-cm LI-RADS 5 lesion, which was treated with stereotactic body radiotherapy (SBRT). The patient ultimately underwent OLT 2 years after the last recurrence. His post-OLT IS regimen included (10 mg) prednisone, (3 mg) tacrolimus, and (500 mg) mycophenolate. Since then, he has been hospitalized twice due to mesh-related complications and infections but has no clinical evidence of rejection.

Patient 7 was a 66-year-old man diagnosed with multifocal HCC and a history of HCV cirrhosis from prior heavy alcohol use. The MRI showed two LI-RADS 5 lesions, a 3.9-cm lesion in segment 6, and a 2.1-cm lesion in segment 7. A follow-up MRI at 6 months later demonstrated tumor growth of the primary lesion and smaller lesions. There was no definitive evidence to suggest metastatic disease; however, further tumor activity was noted in segment 8, after which TACE was performed on the affected segments. The patient underwent W Atezo/Bev and TACE bridging therapy to OLT, showed beneficial anti-tumor response, and met the OLT criteria for 12 months. The patient washed out of ICI therapy for 3 months before proceeding to the OLT procedure. Following the OLT, the patient remained healthy on an IS regimen of mycophenolate and tacrolimus. However, on day 20 post-transplant, moderate acute T-cell mediated rejection was observed, with biopsy revealing mixed portal inflammation, mild lymphocytic cholangitis, and portal venulitis.

Patient 8 was a 74-year-old man diagnosed with stage 2 NASH HCC and cirrhosis at MD Anderson Cancer Center. MRI revealed lesions within the central right hepatic lobe, measuring 3.9 cm, although multiple lesions were noted. Atezo/Bev therapy was initiated, and endoscopy revealed large (>5 mm) esophageal varices with no bleeding, which were treated with beta blockers. At 6 months later, no significant change in hepatic lesions was noted. The patient was also seen by the Houston Methodist transplant team for evaluation. Following an esophagogastroduodenoscopy (EGD) done after 9 months since the diagnosis, the varices were treated with five banding, and bevacizumab was placed on hold, whereas atezolizumab monotherapy was continued. Bevacizumab was resumed 4 months after treatment but paused again for appendectomy. There is no indication of a washout period from atezolizumab, which appears to have been discontinued within a month of OLT. The patient underwent OLT 18 months after being diagnosed and was kept on tacrolimus (1.0 mg) for 1 year. After a year of OLT, the patient had no signs of rejection and was successfully put on a dose of tacrolimus (0.5 mg).

Patient 9 was a 74-year-old man who presented with multifocal hepatic lesions. MRI showed a hypervascular hepatic mass measuring 12.7 cm and encompassing several segments of the liver, with the mass affecting the bile ducts and causing mild biliary dilatation. He was started on Atezo/Bev 3 months following his initial diagnosis and received Y-90 therapy 6 months later. After several cycles, MRI showed that the mass shrunk to 8.6 cm. The Atezo/Bev cycles continued, and additional Y-90 and TARE were used to optimize the treatment and bridge the patient to OLT. A 5-week washout preceded OLT, which was performed a year after diagnosis. Following this procedure, the patient was prescribed mycophenolate mofetil (500 mg), which he took for 3 months before switching to tacrolimus (1 mg), which was further lowered to 0.5 mg in less than a week following the initial intake, but with no specific reason indicated in the patient’s notes. CT and MRI after 1 year of OLT revealed no evidence of recurrent or metastatic disease.

Patient 10 was a 64-year-old man with cirrhosis secondary to alcohol intake and gastric varices (banded). He was confirmed to have end-stage liver disease, verified HCC. Initial MRI showed multiple lesions and unresectable disease. Repeat scans less than 5 months later showed a considerable increase in the size of the mass. CT and MRI imaging demonstrated lesions measuring at least 9.5 × 7.5 cm versus 5.4 × 5.8 cm at the same level as in a prior study. Following successful hepatic angiography, the patient began Y-90 to treat the primary mass and received four cycles of combined ICPI of Durva/Treme. During treatment, the patient presented with upper GI bleeding and acute kidney injury that required hemodialysis, and this resulted in unscheduled pauses to his durvalumab treatment plan. Imaging demonstrated progress in the treated lesions in segment 5 without the development of new lesions. The stable disease allowed the tumor board to evaluate the patient’s proper eligibility for OLT. Subsequently, patient 10 successfully underwent multi-organ transplant of the liver and kidney. The post-transplant patient was placed on mycophenolate mofetil, prednisone, and tacrolimus. A borderline acute rejection of the kidney was observed on day 42 post-transplant, but it was successfully treated. No evidence of rejection of the liver transplant was noted in subsequent follow-up scans.

Patient 11 was a 60-year-old male with NASH cirrhosis, portal HTN with ascites, splenomegaly, and HCC, which was diagnosed as stage III without metastasis. MRI showed two lesions in the right lobe of the liver measuring 4.6 × 3.9 × 5.3 cm and 2.4 × 2.2 × 2.2 cm. A paracentesis was performed, removing 4.3 L of clear yellow fluid. The patient received one cycle of Atezo/Bev at 4 months after diagnosis. His clinical course was complicated by hepatic encephalopathy, portal HTN with ascites, and grade III esophageal varices. He was transferred to HMH for transjugular intrahepatic portosystemic shunt (TIPS) and liver transplant evaluation. After discontinuing immunotherapy for 1 month, the patient underwent OLT. He was placed on an IS regimen of MMF, prednisone, and tacrolimus post-transplant. Follow-up revealed no recurrence or metastasis, and at 1 year post-OLT, there were no indications of rejection or disease recurrence.

Patient 12 was a 38-year-old man with a PMH of congenital biliary atresia; he presented with symptoms of decompensated secondary biliary cirrhosis and elevated AFP levels at 52,000. Imaging showed a right hepatic lesion of 10 cm which was consistent with high-grade neuroendocrine tumor (NET), and the patient received therapy with cisplatin, etoposide, and atezolizumab, followed by SBRT, leading to tumor shrinking. Surveillance imaging revealed disease progression with recurrent masses consistent with HCC and a well to moderately differentiated NET component. Post-OLT pathology confirmed three HCC foci with variable degrees of viability and necrosis with neuroendocrine differentiation and stage 3 to 4 fibrosis. Molecular profiling revealed actionable genetic alterations in ARID1A and CDKN2A. His post-transplant immunosuppressants consisted of tacrolimus, mycophenolate mofetil, prednisone, and everolimus.

Patient 13 was a 73-year-old man who presented with multifocal NASH cirrhosis and moderately differentiated HCC. Initial imaging showed a 13 × 10-cm left liver mass and additional lesions, which progressed to a 16.7-cm left liver mass and a 2.1-cm right liver lesion within a month. He underwent Y-90 therapy to the left lobe and TACE to the right and was started on lenvatinib. At 7 months later, he was removed from the transplant list due to new liver lesions but began liver ablations and received two cycles of Atezo/Bev therapy. He was then able to undergo a successful liver transplant after around 23 months of receiving cancer treatment. He remains on tacrolimus, everolimus, and MMF, with no signs of rejection or recurrence.

Patient 14 was a 33-year-old woman who was diagnosed with fibrolamellar HCC through biopsy, imaging, and positive PRKACA rearrangement. The liver MRI at that time revealed a 10.5 × 7.6-cm mass in segment 4B. Enlargement of another hyper-enhancing nodular area was noted, measuring approximately 2.2 × 2.2 cm. There were few additional similar prominent arterial hyper-enhancing nodular regions along the superior aspect of the mass. At 6 weeks later, the patient was started on gemcitabine and oxaliplatin for eight cycles each, plus a single cycle of lenvatinib. These were followed by nine cycles of 5-fluorouracil (5-FU), peginterferon alfa-2a (Pegasys), and nivolumab to combat the metastatic conditions that were found in follow-up scans, which showed a good response. The patient was subsequently reverted to her initial line of treatment of combined gemcitabine/oxaliplatin (gemox) and completed 14 cycles of therapy. Lenvatinib capsule was also added to the initial cycle of this treatment. After metastasis was found, she had scoping precoder sand and then underwent OLT, which was complicated due to fibrolamellar HCC fistulizing into the duodenum status post Y-90, necessitating a subsequent Whipple procedure. Following surgical intervention, there was no evidence of rejection or any post-op complications, and she then received comprehensive IS including MMF, prednisone, and everolimus.

Patient 15 was a 60-year-old woman with chronic hepatitis C and cirrhosis and who presented with HCC. Imaging showed multiple bilobar liver masses, the largest of which was 6.6 cm, with cystic/necrotic components, a tumor thrombus in the middle hepatic vein, and portal HTN. The patient was staged at IIIB. The patient began Atezo/Bev, resulting in tumor shrinkage and a drop in AFP from 53,000 to <2.7 within 4 months. Treatment was temporarily held due to thrombocytopenia, gingival bleeding, and ascites, which required diuretics and hematology consultation. At 3 months later, imaging showed decreased tumor size, with ascites resolved and no observed metastasis. At 5 months later, the CT scan showed a stable disease. After 12 months of treatment, the patient underwent liver transplantation without complications. Post-transplant, the patient was managed on MMF and tacrolimus for 6 months, with no signs of rejection or recurrence.

Patient 16 was a 63-year-old man with primary liver cancer and who presented with two focal hepatic lesions in the right and left lobes of the liver measuring 5.8 and 2.9 cm, respectively, consistent with HCC. At 2 months later, the lesions progressed with macrovascular invasion into the right portal vein and its branches and distal branch of the middle hepatic vein and staging to IIIA HCC attributed to HCV and metabolic syndrome, with a Child–Pugh score of A. The patient started Atezo/Bev and completed 17 cycles of atezolizumab and 12 cycles of bevacizumab, with a temporary hold on bevacizumab due to XRT treatments but which resumed after 3 months of radiation therapy. After treatment, MRI showed a decrease in size, but the patient was reassessed to stage IIIB. The patient received Y-90 for left hepatic lesion while on Atezo/Bev treatment. After the disease was stabilized, ICPI therapy was discontinued due to tolerance. The patient then underwent OLT and had no signs of transplant rejection. Unfortunately, the patient suffered from post-op complications including respiratory failure, which required ventilator support, and renal failure requiring hemodialysis. He passed away 2 months later due to disease recurrence while still not showing any signs of graft rejection.

Patient 17 was a 71-year-old man with NASH cirrhosis complicated by ascites, esophageal varices, and HCC. MRI revealed multifocal lesions, including the largest lesion that was a 4.6-cm LR5 in segment 7, followed by a 1.6-cm LR5 lesion in segment 4 and with additional lesions in segments 5 and 8. The patient was staged at II and treated with diuretics for ascites and esophageal banding for varices. Due to the history of varices, a combination of Durva/Treme was recommended, and therapy was started; however, tremelimumab was discontinued after one cycle. Durvalumab continued with cycle 3. The patient later developed hepatic encephalopathy and was admitted to the ICU. After management of the complications, the patient underwent OLT after a 1-month washout period. Post-transplant, the patient was started on an IS regimen of cyclosporin (100 mg BID), MMF (500 mg), and prednisone. There were no signs of rejection; however, the patient developed respiratory failure, thus requiring ventilator support. The patient has since shown significant recovery and is now doing well.

Patient 18 was a 36-year-old man who presented with intrahepatic cholangiocarcinoma (IHCCA). A few weeks after his initial diagnosis, he underwent laparoscopy of segment 3 and caudate lobe resection along with portal lymphadenectomy. At 1 month later, gemcitabine, cisplatin, and durvalumab (gem/cis/durva) treatment was initiated. At 2 months following treatment initiation, imaging revealed stable disease in the right lobe with sub-centimeter right diaphragmatic and porta hepatis nodes. MRI of the same regions revealed a 6.3-cm mass in segments 6 and 7 with biliary dilation. The patient then underwent cholecystectomy. A follow-up MRI of the abdomen 2 months later showed a decrease in the size of the right hepatic lobe mass, now measuring 7.0 × 5.0 cm, down from 8.9 × 6.7 cm. By the end of the year, the patient had completed eight rounds of scheduled therapy. The patient then received out-of-state treatment for 7 months, with the last infusion given less than a month before OLT. The patient was also treated with proton therapy for a month at the beginning of that year, which the patient decided to complete after a cycle of SBRT. The patient had immediate graft failure, was rendered anhepatic, and was retransplanted the next day. The patient was put on a structured regimen of mycophenolate mofetil, prednisone, and tacrolimus to support his transition into remission. No acute rejection was evident during the first year post-transplant.

Patient 19 was a 37-year-old man diagnosed with perihilar cholangiocarcinoma (PCCA). The diagnostic lab results demonstrated high LFTs (ALK—1,232, AST—438, and ALT—570), indicating complications of his known history of PSC and liver cirrhosis. MRI revealed no hepatic masses, with further evaluations revealing intrahepatic biliary dilation with mildly distended gallbladder in the fundal region and with a small filling defect in the common bile duct (CBD). An endoscopy in the next 2 months demonstrated multiple diffuse stenoses, with a 2-cm dominant structure at mid-CBD. The main CBD containing diffuse irregularities was successfully dilated. Neoadjuvant ICPI with gem/cis/durva was prescribed, and the patient successfully completed four cycles of treatment prior to OLT, which was done later that year, at around 6.5 months later. There were no signs of rejection, and the patient was placed on a comprehensive IS regimen of prednisone, tacrolimus, and everolimus. The patient had a good recovery following the transplant surgery and was subsequently started on a single agent, gemcitabine, as adjuvant therapy, which was well tolerated.

Patient 20 was a 54-year-old woman diagnosed with locally advanced IHCCA. The initial MRI revealed multiple liver masses (the largest being 10 × 9 × 6 cm) with no metastatic disease. A biopsy confirmed moderate to poorly differentiated adenocarcinoma. Systemic therapy with gem/cis/durva began 2 months after the diagnosis, but Durva was discontinued after three cycles due to grade 3 ICPI-related colitis, which was treated with prednisone. Imaging showed a continued response, and gem/cis was resumed, with a total of nine cycles administered along with Y-90 as bridging therapy. The patient underwent OLT 1 year after treatment initiation. The patient completed seven cycles of adjuvant chemotherapy with gem/cis alongside IS therapy with MMF, prednisone, and tacrolimus. However, elevated LFTs promoted the discontinuation of chemotherapy. There are no signs of transplant rejection or disease recurrence to date.

Patient 21, a 63-year-old man with CCA, initially presented with weight loss, fatigue, and pruritus. Lab evaluation revealed elevated liver enzymes and elevated CA 19–9 levels to 104. The initial CT scans found a 7 × 6.5-cm ill-defined hepatic lesion predominantly in the left lobe with biliary dilatation and occlusion of the left portal vein, and gastrohepatic ligament lymphadenopathy. The biopsy findings were consistent with poorly differentiated adenocarcinoma. Subsequent abdominal MRI showed a 10.7-cm left hepatic mass with mild diffuse biliary ductal dilation and occlusion of the left portal vein and left hepatic artery branches compatible with CCA. At 2 months later, the patient was started on gem/cis/durva and completed 10 cycles of therapy. He then received a cycle of capecitabine with radiation therapy for 5 weeks. The patient resumed maintenance durvalumab treatment afterward. Follow-up imaging showed stable disease until a few months later when A/P the CT scans redemonstrated a hypo-enhancing tumor lesion consistent with disease progression. The patient received once cycle of ivosidenib (250 mg) and was later started on mFOLFOX6 treatment including oxaliplatin, leucovorin, and 5-FU for four cycles. The patient underwent OLT around 5 months later and showed no signs of rejection. The patient was placed on an IS regimen of MMF, prednisone, and tacrolimus.

Patient 22 was a 43-year-old woman who was diagnosed with IHCCA following imaging and biopsy that revealed a 5.4-cm mass in the right hepatic lobe into segments 8 and 4, with extension into the right portal vein, portal hepatic, and peripheral lymph nodes. The patient underwent a right hepatectomy a month later, followed by 6 months of gem/cis chemotherapy. At 3 months later, liver ablation was performed, but CT imaging showed progression of the ablated lesion. The patient subsequently received five cycles of durvalumab and LY3410738 combined with ivosidenib. After that, the patient was placed on futibatinib for 4 months before switching to FOLFOX and subsequently FOLFIRI, which was poorly tolerated. The patient then resumed gemcitabine with abraxane, completing three cycles before holding treatment for Y-90 therapy and later resuming chemotherapy. At 9 months after restarting chemotherapy, the patient underwent OLT and was staged at II, with no signs of rejection, but the patient is still taking MMF, prednisone, and tacrolimus.

Patient 23 was a 45-year-old woman with IHCCA who presented with a 13-cm mass in the left lobe of the liver with separate satellite masses on A/P CT scans. The left focal interventional radiology (IR) biopsy identified moderately differentiated adenocarcinoma. A subsequent PET scan demonstrated an intense uptake to the hepatic mass, reflecting biopsy-proven adenocarcinoma. The patient was started on gem/cis/durva and completed 10 cycles with fever as the only complication. A corresponding PET/CT scan illustrated interval progression of intrahepatic disease with multiple new lesions. Around 5 months later, durvalumab was dropped from her ICPI regimen, and she continuously received two cycles of gemcitabine and cisplatin, with a 2-month washout period prior to OLT. The IS regimen consisted of MMF, prednisone, and tacrolimus. The patient was found to have focal evidence of mild acute T-cell-mediated rejection on day 162 following OLT. She subsequently demonstrated chronic liver rejection on day 206 post-transplant and was relisted for a second OLT.

Patient 24 was a 66-year-old woman with IHCCA. The patient initially presented with fever and was found to have a hepatic abscess; thus, a drain was placed at that time. A month later, magnetic resonance cholangiopancreatography (MRCP) scan was obtained, and it revealed moderate intrahepatic bile duct dilatation due to high-grade stricture at the biliary hilum in addition to a 2.5-cm rim-enhancing fluid collection in segment 8 of the liver consistent with a possible liver abscess. A biopsy was taken from the hilar stricture of the bile duct and was positive for invasive, moderately to poorly differentiated adenocarcinoma. The patient was initiated on gem/cis/durva and completed five cycles of ICPI therapy. Around 14 months later, the patient underwent OLT and showed no signs of rejection while receiving IS therapy with MMF, prednisone, and tacrolimus.

Patient 25 was a 63-year-old man diagnosed with IHCCA after presenting with jaundice and lab abnormalities. The imaging and biopsy results confirmed adenocarcinoma with a 4.8 × 4.6-cm mass at the liver hilum which extended into segments 4 and 8, with biliary dilation and enlarged portacaval nodes. After an initial intervention, including ERCP, stenting, and cholecystostomy, the patient received gem/cis/durva for one cycle and was later switched to FOLFOX for four cycles until disease progression at 5 months after diagnosis. A month later, the patient resumed gem/cis before undergoing OLT. The patient remained stable post-transplant on IS prednisone, everolimus, and tacrolimus without signs of rejection. Due to the extent of the disease, adjuvant capecitabine was initiated a month after transplant and has continued to date.

The treatment details for this study cohort are summarized in **Tables 1** and **2**, and the pathological features that were identified in the explanted liver specimens following OLT are shown in **Table 3**.

**Table 2:** Demographic, clinical and outcome characteristics of patients receiving liver transplantation after immune checkpoint inhibitors.

Case ID	Age, years	Gender	Dx	Type of ICPI	LRT	WOP in months	AFP before OLT	Type of surgery	Rejection	IS agents after OLT
1	66	Male	HCC	PD-L1	SBRT	4	2.0 ng/mL	OLT	No	Mycophenolate, tacrolimus
2	63	Male	HCC	PD-1	Y-90/TACE	1	1.8 ng/mL	OLT	No	Mycophenolate tacrolimus
3	59	Male	HCC	PD-L1	TACE/Y-90	6	4.4 ng/mL	OLT	No	Mycophenolate tacrolimus
4	62	Male	HCC	PD-1, PD-L1	Y-90	2	792.0 ng/mL	OLT	No	Mycophenolate, prednisone, tacrolimus
5	68	Male	HCC	PD-L1	Y-90/RFA/TACE	10	41.2 ng/mL	OLT	No	Mycophenolate, tacrolimus
6	61	Male	HCC	PD-1, CTLA-4	TACE/RFA/SBRT	41	2.7 ng/mL	OLT	No	Mycophenolate, prednisone, tacrolimus
7	66	Male	HCC	PD-L1	TACE	3	14.3 ng/mL	OLT	Yes	Mycophenolate, tacrolimus
8	74	Male	HCC	PD-L1	TACE	1	5.3 ng/mL	OLT	No	Tacrolimus
9	74	Male	HCC	PD-L1	Y-90/TARE	1	1.8 ng/mL	OLT	No	Mycophenolate, tacrolimus
10	64	Male	HCC	PD-L1 CTLA-4	Y-90	2	30.8 ng/mL	OLT	Yes	Mycophenolate, prednisone, tacrolimus
11	60	Male	HCC	PD-L1	None	1	<1.8 ng/mL	OLT	No	Mycophenolate, prednisone, tacrolimus
12	38	Male	HCC	PD-L1	SBRT	32	246.0 ng/mL	OLT	No	Mycophenolate, prednisone, tacrolimus, everolimus
13	73	Male	HCC	PD-L1	Y-90, TACE, ablation	1	54.4 ng/mL	OLT	No	Mycophenolate, tacrolimus, everolimus
14	33	Female	HCC	PD-1	Y-90	16	6.0 ng/mL	OLT	No	Mycophenolate, prednisone, everolimus
15	60	Female	HCC	PD-L1	None	13	2.5 ng/mL	OLT	No	Mycophenolate, tacrolimus
16	62	Male	HCC	PD-L1	XRT Y-90	3	2.4 ng/mL	OLT	No	Mycophenolate, prednisone, tacrolimus
17	71	Male	HCC	PD-L1, CTLA-4	None	1	15.4 ng/mL	OLT	No	Mycophenolate, prednisone, cyclosporin
18	36	Male	IHCCA	PD-L1	SBRT/Proton	0.5	7.8 ng/mL	OLT	IGF	Mycophenolate, prednisone, tacrolimus
19	37	Male	PCCA	PD-L1	None	6.5	3.3 ng/mL	OLT	No	Prednisone, tacrolimus, everolimus
20	54	Female	IHCCA	PD-L1	Y90	10	5.7 ng/mL	OLT	No	Mycophenolate, prednisone, tacrolimus
21	63	Male	IHCCA	PD-L1	XRT	16	3.1 ng/mL	OLT	No	Mycophenolate, prednisone, tacrolimus
22	43	Female	IHCCA	PD-L1	Ablation, Y-90, TACE	18	5.2 ng/mL	OLT	No	Mycophenolate, prednisone, tacrolimus
23	45	Female	IHCCA	PD-L1	None	2	2.7 ng/mL	OLT	Yes	Mycophenolate, prednisone, tacrolimus
24	66	Female	IHCCA	PD-L1	None	14	17.5 ng/mL	OLT	No	Mycophenolate, prednisone, tacrolimus
25	63	Male	IHCCA	PD-L1	None	5	2 ng/mL	OLT	No	Prednisone, tacrolimus, everolimus

AFP, alpha-fetoprotein; CTL-4, cytotoxic T-lymphocyte–associated antigen 4; Dx, diagnosis; HCC, hepatocellular carcinoma; ICPI, immune checkpoint inhibitors; IGF, immediate graft failure; IS, immunosuppressant; LRT, locoregional therapy; ng/mL, nanograms per milliliter; OLT, orthotopic liver transplantation; PD-1, programmed death; PDL-1, programmed death-ligand 1; RFA, radiofrequency ablation; TACE, transarterial chemoembolization; WOP, washout period; XRT, external radiation therapy; Y90, yttrium-90.

**Table 3:** Pathological characteristics of explanted specimens post-OLT following locoregional treatment and immunotherapy regimens.

Case ID	Tumor size (cm)	Tumor focality	Hepatic segment(s) involved by HCC	Grade	Tumor necrosis	Lymphovascular invasion	Perineural invasion	Primary tumor (ypT)	Lymph nodes	Tumor stage	Etiology associated with HCC/CCA	Fibrosis staging
1	3.3, 2.9, 1.2	Multifocal, 3 tumor nodules	2, 2, and 7	Poorly differentiated	25%	Not identified	Not identified	T2	One (ypN0)	II	Hepatitis B and C	Cirrhosis
2	0.4	Unifocal	4	Well-differentiated	99%	Not identified	Not identified	T1a	None	IA	Hepatitis C, alpha-1 antitrypsin deficiency	Cirrhosis
3	0.1–2.7	Multifocal (cirrhotomimetic) >30 tumor nodules	All segments	Moderately differentiated	20%	Present, small-caliber blood vessels	Not identified	T2	None	II	Hepatitis B	Bridging (stage 3) fibrosis
4	1.2–3	Multifocal, 8 tumor nodules	2, 3, 4, 7, and 8	Moderately to poorly differentiated	4 entirely viable tumor nodules, remainder 4 nodules with 10%–95% necrosis	Present, small-caliber blood vessels	Present, large- caliber nerve fibers	T2	None	II	NASH, hemochromatosis	Cirrhosis
5	8, 5.2, 2.5	Multifocal, 3 tumor nodules	5/6, 2/4, and 7/8	Moderately to poorly differentiated	98% (largest tumor), 70% (second largest tumor), 100% (smallest tumor)	Present, small-and medium-caliber blood vessels	Not identified	T3	Two (ypN0)	IIIA	NASH	Cirrhosis
6	3.4	Unifocal	Perihilar	Well to moderately differentiated	Not identified	Present, small-caliber blood vessels	Not identified	T2	None	II	Hepatitis B	Cirrhosis with regression and portal vein thrombosis in 0egment 2
7	1.5, 1.5, 0.8, 0.7, 0.7	Multifocal, 5 tumor nodules	3 and 7	Moderately differentiated	100% necrosis (first and second nodules)	Not identified	Not identified	T2	None	II	Hepatitis C	Cirrhosis
8	3.8, 1.7, 2, 1	Multifocal, 5 tumor nodules	8 and 7/8	Well differentiated	5%, 30%, 20% (first and last nodules)	Not identified	Not identified	T2	None	II	NASH	Cirrhosis
9	0 (complete response)	Unifocal	4 and 8	N/A	100% Necrosis in Tumor bed	Not identified	Not identified	ypT0	None	0	Hepatitis C	Cirrhosis
10	0.3, 3.4	Multifocal	Involving entire liver	Well to Moderately differentiated	Not identified	Small caliber blood vessels	Not identified	ypT2	None	II	NASH	Cirrhosis
11	6, 2.2	Multifocal	3 and 4	Moderately differentiated	Not identified	Not identified	Not identified	pT3	None	III	NASH	Cirrhosis
12	5.5,4.2,2.4	Multifocal, 3 tumor nodules	6–8	Well to moderately differentiated	10% and 20% (first and second nodules)	Not identified	Not identified	ypT3	None	IIIA	Secondary biliary cirrhosis	Stage 3–4 fibrosis
13	9.9,6.1,5.1	Multifocal	Involving entire liver	Moderately differentiated	70%	Not identified	Not identified	pT3	None	IIIA	NASH	Cirrhosis
14	14.8, 2.5, 1.7, 0.7	Multifocal	2, 3, 4, 7	N/A	Extensive necrosis	Not identified	Not identified	pT4	None	IIIB	PRKACA gene rearrangements	Cirrhosis
15	0	Multifocal	4, 5, 6, 7, 8	N/A	100%	Not identified	Not identified	ypT0	None	0	Hepatitis C	Cirrhosis
16	2.9, 0.7, 1.9, 1.8, 0.7	Multifocal	2 (two lesions), 4, 5, 8	Poorly differentiated	70%, 5%, 5%, 80%, 90%, 70% (segments 2, 2, 4, 5, 8)	Large and small vessel invasion including portal venous branch	Present	pT4	None	IIIB	Hepatitis C	Cirrhosis
17	0.3	Multifocal, 3	7, 4 Lt/Rt lobe	Moderately differentiated	Not identified	Not identified	Not identified	pT2	None	II	ETOH	Cirrhosis
18	7.5	Unifocal	IHCCA 7	Moderately differentiated	80- 90%	Small vessels invasion	Present	ypT2	None	II	N/A	N/A
19	Thickening/dilation	Unifocal	PCCA 1, 2, 4	Well differentiated	Not identified	Not identified	Not identified	T1	None	I	Primary sclerosing cholangitis (PSC)	Stage 3 fibrosis
20	10.5, 4, 0.7	Multifocal	IHCCA 4, 5, 6, 2	Moderately to poorly differentiated	40%	Not identified	Not identified	ypT2	None	IIIA	N/A	N/A
21	7.3	Unifocal	IHCCA 2,3	Moderately to poorly differentiated	100%	Present	Not identified	ypT0	1	yIIIB	Chronic cholecystitis	Stage 3–4 cirrhosis
22	5.8	Unifocal	IHCCA 4, 8	Moderately differentiated	Not identified	Not identified	Not identified	ypT1b	None	IB	N/A	N/A
23	15, 1.3, 1.8, 1.1, 0.8	Multifocal	IHCCA 2, 3, 4, 5, 9	Moderately differentiated	20%	Suspicious foci identified	Not identified	ypT2	None	II	N/A	Minimal macrovesicular steatosis
24	10.5	Unifocal	IHCCA of the right lobe	Moderately differentiated	N/A	Present	Not identified	pT2aN1	Three	IVa	Mild chronic cholecystitis	Periportal chronic inflammation 40% macrovesicular steatosis and septal fibrosis
25	7, 1.2	Multifocal	IHCCA 4, 8	Moderately differentiated	Not identified	Not identified	Present	ypT2	None	II	N/A	N/A

## 4 Discussion

The study included 25 patients who received different regimens of ICPI therapy in various combination forms with non-uniform LRT interventions. During the first year post-transplant, four patients experienced graft-related complications. One patient developed immediate graft failure on the day of transplant, requiring emergent anhepatic status and successful retransplantation on the following day. The remaining three experienced acute rejection: two involving the liver allograft and one involving a deceased donor kidney that had been transplanted concurrently with OLT. A combination therapy of atezo/bev was administered to 11 patients in this series. Patients 1 and 4, in this report, received neoadjuvant therapy combining atezo/bev for HCC at stages II and I, respectively. Treatment was discontinued in preparation for OLT, which occurred 4 and 3 months after the last immunotherapy administration. Patient 5, diagnosed with stage III HCC, commenced treatment with a combination of atezo/bev. The significant advancement of the disease necessitated a transition to an alternative FDA-approved treatment—lenvatinib, which was discontinued 5 months before the OLT. In the case of patient 3, diagnosed with stage II HCC, the commencement of atezo/bev therapy led to disease progression. The treatment was succeeded by a 4-month course of targeted therapy using cabozantinib prior to OLT.

The expansion of this series, which prompted this publication, can be identified in patients 7–25. There are several similarities that can be seen in the almost standard utilization of atezo/bev, but there is a much more expansive utilization of gem/cis/durva as a combination ICPI intervention. This can, in some form, be traced back to clinicians following the methodology of the TOPAZ-1 study, which attempted to mimic the beneficial survival outcomes that were reported (33). Some of these patients showed more extreme complications and variations.

Patients 13, 15, and 16 received a combination treatment of ICPI with atezo/bev. A significant finding that may reflect the importance of ICPI use before OLT was seen in patients 13 and 16, whose imaging showed a decrease in the size of their hepatic lesions and stabilization of the disease, enabling subsequent eligibility for OLT. In addition, patient 13 was initially removed from the transplant waiting list due to disease progression. However, following liver ablation therapy with TACE and Y-90 and receiving two cycles of atezo/bev, he was successfully relisted and eligible for liver transplant. Another remarkable finding was seen in patient 15 who had an initial AFP of 53,000 ng/mL that dropped to less than 2.7 ng/mL following 4 months of treatment with atezo/bev. Patient 14 received nine cycles of nivolumab with 5-FU and peginterferon alfa-2a, had a good response to therapy, and then underwent OLT with no signs of graft rejection. Patients 10 and 17 had HCC and received ICPI treatment with Durva/Treme for four and three cycles, respectively. Both patients successfully achieved stable disease and underwent OLT within 2 and 1 month of stopping ICPI therapy, respectively. Eight patients with CCA received ICPI treatment combined with gem/cis/durva. Notably, patient 18 demonstrated a decrease in the size of the liver lesion and achieved a stable disease response before undergoing OLT. He subsequently had immediate graft failure, was made anhepatic, and retransplanted the following day.

This study highlighted that those patients with unifocal lesions who utilized ICPI had better outcomes than those with multifocal lesions. Most patients in our study cohort received LRT with either TACE, Y-90, SBRT, or XRT. Notably, the patients who received LRT were shown to have increased tumor control by facilitating tumor downstaging and enhancing transplant eligibility. In contrast, the seven patients who did not receive LRT relied completely on ICPI therapy, which resulted in less consistent tumor reduction and stabilization in some cases compared to those who received combination therapy of both LRT and ICPI. Our study demonstrated that most patients received ICPI prior to OLT without significant adverse effects. However, patient 20 developed grade 3 ICPI-related colitis, which was successfully managed with prednisone.

Following OLT, most patients showed good recovery with no complications, except for four patients who died. Patient 1 experienced a caval thrombus and died on day 342 after finding HCC recurrence on day 221. Patient 3 experienced HCC recurrence on day 361 and died on day 699 from diffuse multifocal HCC with metastasis. Patient 5 died of multisystem organ failure on day 66 post-transplant. Patient 16 had disease recurrence and devolved respiratory and renal failure, required ventilator support and hemodialysis, and ultimately passed away on day 61 post-transplant. Moreover, patient 17 experienced respiratory failure and required ventilator support, after which he had significant recovery. Collectively, four patients in our cohort experienced graft complications within the first year post-transplant, including one case of immediate graft failure requiring retransplantation and three cases of acute rejection involving either the liver or a concurrently transplanted kidney allograft.

This study examines the safety of immunotherapy agents, including atezolizumab, nivolumab, and ipilimumab, administered prior to OLT, highlighting the significance of the timing of ICPI administration in relation to OLT. A previously published cohort of eight patients who underwent ICPIs followed by OLT reported an interval of 4 weeks between the last ICPI dose and OLT, with only one instance of mild biopsy-proven acute rejection (BPAR) (13). The risk of acute rejection may be heightened, particularly with a brief washout period between the final administration of ICPIs and OLT (14). The cases indicate that an extended duration without immunotherapy before orthotopic liver transplantation may enhance transplant outcomes in patients with a history of immunotherapy. The precise duration of washout prior to OLT remains unverified, with estimates varying from 4 to 12 weeks (12–15). Mazzaferro et al. recently published a comprehensive review of the evolution to HCC care, particularly in the context of transplant oncology, which states that an optimal washout period before transplantation is 30 days. Based on our institutional experience, a 4-week period between the final dose of ICPIs and OLT is recommended to mitigate the risk of significant liver-related complications, including liver allograft rejection or toxicity, though, admittedly, this study does showcase some patients receiving a washout less than the recommended interval with no current signs of graft rejection. Any reinforcement of a minimum period would be difficult, as it is not always feasible to align with the schedule of viable organ availability, though it does work to ensure the smallest risk of rejection or toxicity.

Patients who are naïve to ICPI and are being studied for tumor response and bridging capacity would be the optimal target for clinical analysis. Following these observations, a greater variety of prospective clinical trials are necessary to optimize the use of ICPIs in patients waiting for OLTs, enhance risk predication, and decrease the rate of graft loss (34). Future research should prospectively investigate the differences between patients who received prior treatment before ICPI and those for whom immunotherapy was the initial intervention. Immunotherapy can induce significant graft rejection in certain patients through the activation of the innate immune response. Of course, it would also be beneficial to increase education and availability of prevention measures for these diseases, such as encouraging annual physicals or promoting greater emphasis on nutrition for overall health.

As it stands, immunotherapy is quickly being standardized as a treatment modality that yields promising prognostic outcomes in patients with HBC. In the US, this is, at least in part, due to the rate of approval of these ICPI treatments by the FDA. While current treatments have improved and are constantly promoting and innovating to provide better progression-free survival (PFS) and OS, a significant proportion of patients, up to one-third, do not experience substantial benefits from this approach, and immune-related adverse events may occur in up to one-fourth of these cases (35). It is currently difficult to predict which patients will benefit from ICPI therapy based on clinical and pathological parameters. There is a lack of novel predictive biomarkers that can assist in evaluating responses to immunotherapy and inform subsequent clinical treatment strategies. Studies have shown a strong correlation between the levels of serological laboratory markers, such as AFP, and inflammatory markers, such as C-reactive protein, with patient prognosis in advanced HCC cases (36, 37). It is encouraging that there are emerging techniques, such as molecular testing and ctDNA, to monitor diagnostic values and disease mutations as well as better surveillance methods to assess recurrence (38, 39).

The integration of LRT and immunotherapy led to substantial tumor necrosis, varying from 10% to 100% in all cases except in three patients (**Table 3**). The application of response criteria aims to identify patients who may benefit from continuing their current treatment versus those who might gain more from alternative treatments, particularly in terms of improved OS outcomes. This study indicates that combining neoadjuvant immunotherapy and LRT with OLT in the management of advanced HBC effectively reduces the volume of viable tumors and offers promising outcomes without compromising transplant feasibility or safety.

This study, although retrospective and limited by the number of patients, underscores the importance of timing for ICPI administration prior to OLT and highlights the necessity of an appropriate washout period to reduce the risk of acute rejection. Future cohort studies conducted prospectively with rigorous selection criteria are essential to demonstrate the efficacy of ICPI agents prior to OLT. This study advocates for the identification of appropriate predictive biomarkers to enhance patient selection criteria for neoadjuvant ICPI therapy in hepatobiliary patients within an OLT context.

## 5 Conclusions

Our experience emphasizes the significant role of ICPIs in achieving tumor downstaging prior to OLT when employed as a sole or first-line therapeutic strategy in patients with HBC. In addition, this study highlights the importance of the timing of ICPI administration before organ transplantation while supporting the safety of immunotherapy in the pre-transplant setting. Although the optimal timing of ICPI administration prior to transplantation remains a subject of ongoing debate and warrants further investigation, our institutional experience suggests that a minimum interval of approximately 4 weeks may be associated with favorable outcomes. Given the limited and inconsistent data available in this therapeutic area, we believe that our study provides a foundation for future prospective trials evaluating the impact of CTLA-4 and PD-1/PD-L1 inhibitors in patients undergoing liver transplantation.

## Data Availability

The raw data supporting the conclusions of this article will be made available by the authors, without undue reservation.
